# 

**DOI:** 10.1192/bjb.2025.19

**Published:** 2026-02

**Authors:** Jonathan Green

**Affiliations:** Professor of Child and Adolescent Psychiatry, University of Manchester, UK; Royal Manchester Children’s Hospital, Manchester, UK

**Figure:**
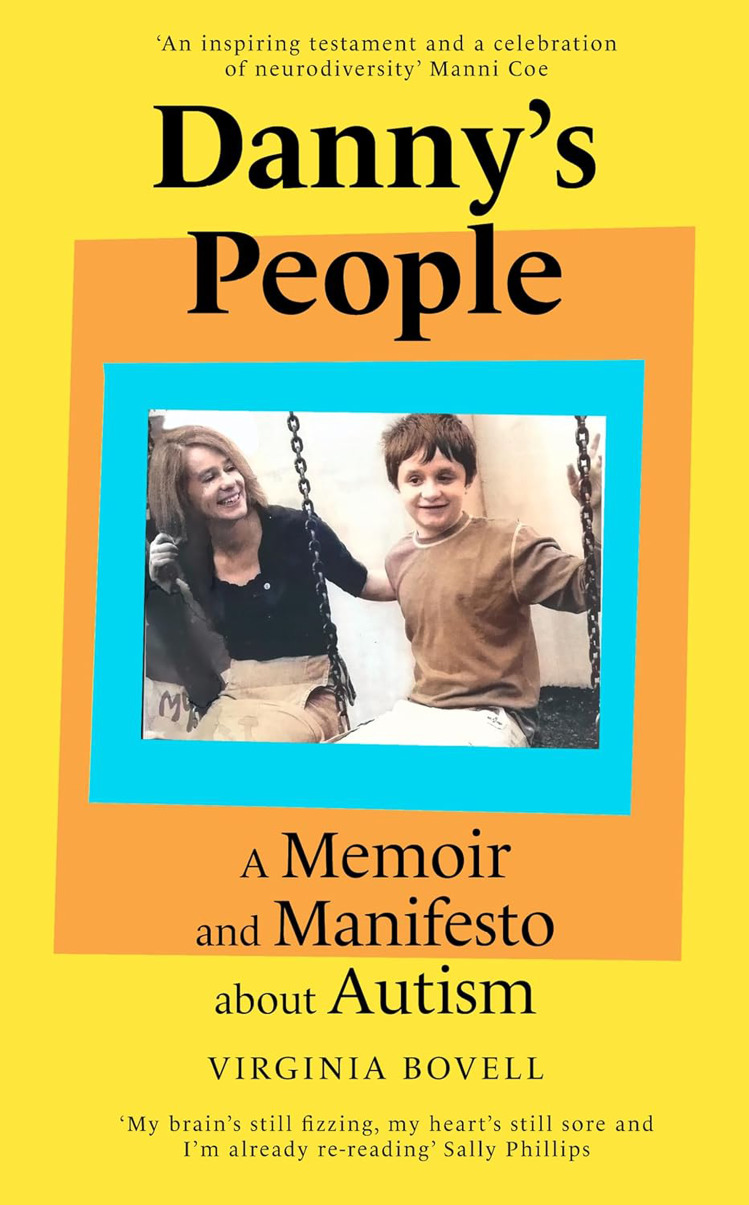

As clinicians, we become deeply engaged with the parents and children who come to us. Virginia Bovell’s book takes us through the looking glass to the other side of such roles: into the lived experience of a mother and family adapting to neurodiversity and, within that, interacting with clinicians like us.

Her book covers 30 years from the birth of her son Danny in 1993 to the present, a period that roughly corresponds to my own clinical lifespan. It has been an extraordinary period of rapidly shifting scientific understanding and social attitudes in relation to autism. She experiences all this with her family as a parent as Danny grows, but also increasingly as a writer and campaigner, engaging with the social, medical and science cultures as they evolve – and, in the end, taking a doctorate.

This combination makes for a remarkable memoir. She strives to be even-handed, but increasingly expresses outrage at what is not there for her child. She begins to campaign with friends and family to build the kind of personal and educational environment that they feel Danny needs, which eventually becomes the Tree House school and charity in London.

There are some key moments with psychiatry, and hours in A&E and surgery for Danny’s complex, late-diagnosed bowel problems. Generally, however, medicine is at the periphery of awareness; the foreground focus is on family and community everyday. ‘Danny’s people’ are largely these people, showing that amazing concentration of parental and wider community care so often seen in response to a child’s enduring special needs.

On this turns the ‘manifesto’ in the book, weaving its way through the narrative alongside her parental experience. Simply put, what is the value of a life, different from the normal, containing more than a usual amount of everyday suffering but with highlights of joy? The value not only to parents and close family, but to wider society? A life that is not ‘productive’ in a conventional sense, indeed a life that consumes more than a usual amount of resource for its stability? What is our metric for valuing such a life and non-normative difference? These are unsentimental and pressing questions, often about simple attention and resource allocation within a society geared towards maximal productivity and monetary efficiency. They are not easy or straightforward, since the resources needed can be significant and it is an area with insufficient clinicians. How does this sort of life and need compare with the resource needed for other illnesses, not to mention other social costs, such as, as she says, warfare? Virginia Bovell asks us to consider these questions, arguing for the value of the life that she shares with her child.

We see these things every day, as clinicians, from the perspective of our professional mission and roles. Virginia Bovell takes us vividly to the other side of that glass, looking back at ourselves in a startling way, critically sometimes but hopefully too … in a way that distils a question: what, at the core, in this context, is needed from us?

